# Understanding nurses’ dual practice: a scoping review of what we know and what we still need to ask on nurses holding multiple jobs

**DOI:** 10.1186/s12960-018-0276-x

**Published:** 2018-02-22

**Authors:** Giuliano Russo, Inês Fronteira, Tiago Silva Jesus, James Buchan

**Affiliations:** 1 Centre for Primary Care and Public Health, Queen Mary University of London, Yvonne Carter Building, 58 Turner Street, London, E1 2AB United Kingdom; 2 GHTM, Instituto de Higiene e Medicina Tropical, Nova University of Lisbon, Rua da Junqueira 100, Lisbon, Portugal; 3 School of Health Sciences, Queen Margaret University, Edinburgh, EH21 6UU United Kingdom

**Keywords:** Nurses dual practice, Multiple job-holding, Moonlighting, Human resources for health, Private health sector, Nurses, Casualization of work

## Abstract

**Background:**

Mounting evidence suggests that holding multiple concurrent jobs in public and private (dual practice) is common among health workers in low- as well as high-income countries. Nurses are world’s largest health professional workforce and a critical resource for achieving Universal Health Coverage. Nonetheless, little is known about nurses’ engagement with dual practice.

**Methods:**

We conducted a scoping review of the literature on nurses’ dual practice with the objective of generating hypotheses on its nature and consequences, and define a research agenda on the phenomenon. The Arksey and O’Malley’s methodological steps were followed to develop the research questions, identify relevant studies, include/exclude studies, extract the data, and report the findings. PRISMA guidelines were additionally used to conduct the review and report on results.

**Results:**

Of the initial 194 records identified, a total of 35 met the inclusion criteria for nurses’ dual practice; the vast majority (65%) were peer-reviewed publications, followed by nursing magazine publications (19%), reports, and doctoral dissertations. Twenty publications focused on high-income countries, 16 on low- or middle-income ones, and two had a multi country perspective.

Although holding multiple jobs not always amounted to dual practice, several ways were found for public-sector nurses to engage concomitantly in public and private employments, in regulated as well as in informal, casual fashions. Some of these forms were reported as particularly prevalent, from over 50% in Australia, Canada, and the UK, to 28% in South Africa. The opportunity to increase a meagre salary, but also a dissatisfaction with the main job and the flexibility offered by multiple job-holding arrangements, were among the reported reasons for engaging in these practices.

**Discussion and conclusions:**

Limited and mostly circumstantial evidence exists on nurses’ dual practice, with the few existing studies suggesting that the phenomenon is likely to be very common and carry  implications for health systems and nurses’ welfare worldwide. We offer an agenda for future research to consolidate the existing evidence and to further explore nurses’ motivation; without a better understanding of nurse dual practice, this will continue to be a largely ‘hidden’ element in nursing workforce policy and practice, with an unclear impact on the delivery of care.

**Electronic supplementary material:**

The online version of this article (10.1186/s12960-018-0276-x) contains supplementary material, which is available to authorized users.

## Background

Health workers’ dual practice has been identified as one of the priority research areas in the human resources for health domain [1]. There is a concern among policy-makers and patients alike that simultaneous engagement with public and private sector activities jeopardise the availability of professionals and the quality of services in the public sector and divert patients towards costlier private care, therefore putting at risk the attainment of Universal Health Coverage (UHC) goals [2, 3].

‘Dual practice’ in the health sector has been defined as health workers’ concomitant engagement in public and private sector clinical activities, with the public sector job representing the ‘primary’ one to which the largest proportion of working hours are allocated [4]. Although very common worldwide, the practice has been traditionally treated with suspicion by the public health and health system research literature, amid fears that it may compromise the supply of public services [5] and encourage absenteeism in public institutions [6], as well as the selection and diversion of patients towards private services [7]. Scholars have highlighted the possible potential benefits of the practice, such as the opportunity it offers to provide a wider range of health services to the population and to retain underpaid workers in the public sector [8]. Others have paid attention to the regulatory aspects [9], with some focusing on the systems’ governance and institutions [10] and others on the incentives to be offered to achieve the desired level of service provision [11, 12].

Substantial literature exists on physicians’ dual practice [13, 14], most recently building up evidence on its prevalence, forms, and drivers worldwide [15–17], as well as on modelling possible regulatory frameworks [12, 18]. However, nurses’ engagement in multiple job-holding is, in comparison, less explored, despite preliminary evidence of its high prevalence in high-income [19] as well as in low-income settings [20], and amid concerns of its impact on the nurses’ wellbeing [21].

Nurses and midwives are the world's largest group of health professionals, representing 48% of the global health workforce, and their role is widely considered critical for the delivery of UHC goals in high- as well as low-income countries [22]. However, the profession has recently come under pressure because of growth of the demand for health services and concomitant scarcity of funds, and the global shifts in the world’s health labour market [23]. As the nursing workforce is predominately female, policy options to address nurses’ participation in the public and private labour market will need to take gender into account [24, 25].

This scoping review sets out to fill this knowledge gap by systematically searching and reviewing the studies conducted on nurses’ simultaneous engagement in public and private clinical activities [26]. Its specific objectives are (1) to map out the existing literature on the subject, determining its prevalence and distribution across geographies, publication types (e.g. peer-reviewed, grey), and specific topics addressed; (2) summarise the evidence, perspectives, and specific contents addressed; and (3) propose an agenda to advance research and development activities to first identify and then mitigate any pervasive effects of nurses’ dual practices to UHC, based on the scoping review results.

## Methods

A scoping review was conducted to determine the extent and key themes within the literature on nurses’ dual practice, as well as to identify areas for future research on the topic. Such knowledge synthesis method is commonly used to address exploratory research questions, to map the existing literature on a field or to preliminarily identify gaps in that literature [26–28]. We used the five Arksey and O’Malley’s methodological steps to develop the research questions, identify relevant studies, include/exclude articles, extract the data, and report the findings [28]. As the methodological guidance for the report of scoping review is still under development, we used the PRISMA guidelines where appropriate [29].

In March 2017, we searched MEDLINE (through PubMed), the ISI Web of Knowledge, Scopus, and the CINHAL Plus with full texts (through EBSCO). We used a set of keywords for the searches and, where appropriate, Medical Subject Headings for nurses combined with keywords and indexed terms related to dual practice (using the Bolean operator ‘AND’). Additional file 1 provides full details for the initial search strategy for each of the databases searched. The grey literature also was searched by visiting websites dedicated to nursing and/or health workforce issues. To widen the scope of the review, the searches on databases and grey literature were not filtered for publication date, language, or publication type. Human resources for health experts (named in the Additional file 1) were a priori contacted to provide relevant references on forms of dual practices among nurses. A posteriori (early December 2017), and based on suggestions coming from the peer-review process, we expand the search terms in the database searches (adding the keywords ‘temporary employment’ and ‘multiple employers’) to provide a few additional records which were considered for the review results, as well. Iterative rather than strictly streamlined procedures are typical in the process of conducting scoping reviews [26]. A final search strategy included snowballing searches (reference list scanning, author tracking) performed on the articles preliminarily selected. References from databases or other sources were filtered through the same eligibility criteria.

To be included, studies needed to address explicitly both nurses and dual practice issues. The working definition for the ‘nurses’ category contained explicit reference in the text to the professional label, with midwives included too. The working definition of dual practice, in turn, referred to concomitant practice in two (or more) distinct clinical services, either in the same or in different healthcare institutions. Public employment was considered the primary job, whereas the secondary (or subsequent) job(s) was considered the one(s) where fewer working hours were spent, periodically or regularly. Alternative labels for dual practice included ‘moonlighting’, ‘public-private work’, ‘multiple profit-generating activities’, ‘dual/multiple job-holding’, and ‘second jobs’. ‘Casualization of work’, defined as the process of replacing full-time and regular part-time staff with contract staff employed on an ad hoc basis, is another phenomenon related in many ways to dual practice [30]. As such, papers addressing this form of employment in relation to dual practice were also included.

Documents in English, French, Portuguese, Italian, and Spanish were included. With the exception of journal commentaries, editorials, and letters to the editor, we did not exclude references because of the type of article (such as opinion pieces), study output (e.g. final or preliminary results), countries or world regions, publication status (i.e. both peer-reviewed and grey literature), or publication date. Titles and abstracts were first screened by one of the authors (TJ) and then reviewed in duplicate by the first author (GR), who finally determined the suitability for the full-text review. Full-text review was carried out by one of three authors, all with a research track record in nurse workforce and/or dual practice issues (GR, IF, JB). Any of the authors were able to directly include or exclude papers on the basis of the eligibility criteria; agreement between two or more reviewers was sought for doubtful cases.

Based on the overarching aim of the paper, the preliminary knowledge of the literature, and a priori consultation with health professionals [13], we developed the following set of questions to guide the data extraction for the review:

- What are the forms in which nurses engage in multiple profit-generating activities?
- What are the different features of nurses’ multiple job-holding?
- What is the prevalence of this phenomenon in nursing?
- Why do nurses engage in dual practice?
- What are the enablers and barriers for nurses’ dual practice?
- What are the personal/ professional drivers and consequences?
- What are the consequences for health systems, specifically for the delivery of quality and safe nursing/health care?
- What are the consequences for nurses’ welfare?
- What are the consequences for patients?
- What are health workers’, managers’, and patients’ perceptions around this practice?

Data extraction tables were then purposively built by the research team to collect data on the specific questions above, either using textual data or synthesis of the articles’ findings/conclusions. Consistent with the scoping review methodology, the data extraction did not involve quality appraisal or grading of the evidence from the studies.

A conventional form of qualitative content analysis, with coding categories derived directly from the text data, was used to analyse data retrieved for each topic [31]. The first author performed a first synthesis of the extracted material, that was then iteratively edited by two of the other authors (IF, JB) following the themes from the data extraction table.

## Findings from the literature review on nurses’ dual practice

**Fig. 1:**
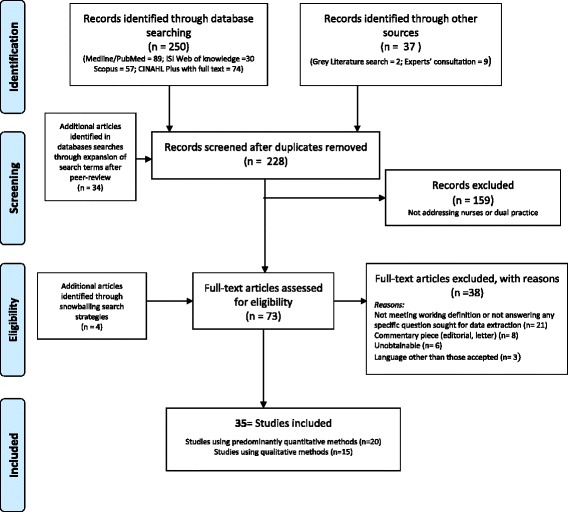
Flow diagram of the scoping literature review process

From 228 records retrieved, 159 (70%) were excluded after reviewing their titles and abstracts (Fig. 1). An additional four articles were identified through snowballing search strategies, resulting in a total of 73 full texts assessed for eligibility. Of all these, a total of 35 (48%) articles finally met the inclusion criteria for addressing dual practices of nurses: 20 using predominantly quantitative methods and 15 using mostly qualitative designs. The vast majority of the studies were in English, with only four published in Portuguese and one in Spanish. Additional file 2 provides spreadsheets for the (a) list of included articles organised by study-type, (b) the data extraction table, and (c) list of articles excluded with the respective reasons.

The vast majority of such documents (65%) were peer-reviewed publications, with the remainder being nursing magazine publications (19%), reports, and doctoral dissertations. Twenty publications focused on high-income countries (particularly on the USA, UK, Canada, and Australia), 16 on low- and middle-income ones (South Africa, Ethiopia, Iran, and Uganda), and two provided a global view on the phenomenon. Many of the documents (*n* = 28) reported information on the prevalence of the phenomenon, and discussed its different forms (25). Drivers and motivations of nurses’ multiple job-holding were the subject of 10 (out of 38) of the documents, while individual and institutional consequences of the practice were discussed in 9 and 12 pieces, respectively. Only seven of the retrieved documents mentioned policy options associated with nurses’ multiple-job holding. Below we present the literature retrieved, organised in sections reflecting the emerging themes.

### Forms of nurses’ multiple job-holding

From the documents retrieved, it emerged that nurses’ engagement in dual practice can take different forms and shapes, with often blurred boundaries. Some authors mention ‘secondary jobs’ and ‘moonlighting’ practices, where public sector nurses engage with the private sector either individually or through an organised nursing services agency [32–34]. Ribera Silva et al. (2009) as well as Gupta et al. (2006) refer generally to ‘nurses taking up public or private secondary jobs’ in Brazil, Chad, Côte d’Ivoire, Zimbabwe, and Mozambique. A similar operational definition is adopted by Serra et al. to describe nurses’ practicing simultaneously in the national healthcare system and for NGOs or private clinics. Publications from HICs at times use the expression ‘casualization of work’ to describe job insecurity through a lack of a stable contract of employment, but also the practice of working flexibly for public and private health facilities, often through agencies and banks for outsourced nursing services [35–38].

However, a distinction is drawn in the literature between holding multiple jobs concurrently, and dual practice, where the nurse’s primary job is in the public sector, and that may be affected in many forms by the simultaneous engagement with other clinical, profit-generating activities [13]. Three common forms of nurses’ dual practice are mentioned, and often used interchangeably, in the literature;

- Primary public sector employment with additional nursing work in the public sector—typically nightly extra shifts in different departments of the same hospital/facility, or other public facilities in the same geographical area [39, 40];
- Primary public sector employment with additional nursing work in the formal or informal private sector—long-hours shifts, or side jobs during spare time/vacation from main employment [34, 41, 42];
- Fixed part-time employment in the public, coupled with multiple flexible contract assignments in public and private sector, often though nurse agencies (referred to as ‘casualization’) [21, 43, 44].

Some authors report that boundaries between public and private sector employment are often blurred, particularly in low-income settings, and that ad hoc classifications of multiple-job holding may be required to capture the essence of the practice for specific countries [2].

### Magnitude of the phenomenon

Although using different definitions of the practice, a number of studies attempted measuring the prevalence of nurses’ engagement in multiple job-holding in high-income as well as low- and middle-income countries (see Table 1 below). These are mainly cross-sectional surveys that do not provide data on trends for the phenomenon.

**Table 1:** Documents retrieved, by key themes

Author and year	Magnitude	Forms	Specific settings/examples	Drivers and motivations	Consequences for provision of services	Consequences for nurses’ health	Policy options
Aiken (2007) [58]	Review of US nurse supply and demand, trends in nurse immigration	Does not specifically examine dual practice					
Alameddin et al. (2009a) [59]	Examines impact of ‘just in time’ staffing policy in Ontario, Canada-and SARS outbreak; interviews with 13 nurse administrators.	Examines impact of ‘just in time’ staffing policy in Ontario, Canada- fewer full time staff, more part time and casual staff and agency staff.			Reports higher costs, reduced surge capacity	Fewer staff meant more overtime, stress related absenteeism increased.	
Alameddin et al. (2009b) [48]	Tracking of 201,463 nurses registered with College of Ontario, 1993–2004	Focus is on all types of job, career move. Limited examination of casualisation					
Batch and Windsor (2009) [60]	(a) Nursing has a higher rate of casualization than other professional and highly skilled workforces; (b) In Australia in 2011 in 2011, 47.5% of nurses were employed in non-standard work.	Casualisation of work	Australian hospitals	The casualisation movement aims at creating a more flexible and cheaper nurse workforce		Part-time and casual nurses are marginalised and excluded - called hole-pluggers; Marginalisation is accepted and normalised as a trade-off for the flexibility of this modality	
Batch et al. (2015) [44]	In 2007 the percentage of part-time and casual nurses, that is those nurses working less than 35 h per week, is 49.8% or almost half the nursing population			Inflexible rosters and unreasonable and unreasonable workloads, unavailability of full-time work in the area of choice. Lack of opportunities for professional development.	Casual and part-time workers seen as peripheral workforce; Marginalisation of casual workers with consequences in career advancement; Implications in the quality of care due to lack of continuity of care and poor match of nurses’ skills to a workplace.		
Baumann et al. (2006) [30]		‘Casualisation’ of the health workforce in Ontario following the SARS epidemic.	Canada (Ontario).		Employing an unbalanced proportion of full time and casual nurses reduces flexibility of a hospital management, as these latter would be less available to cover for unforeseen needs		
Bhengu (2001) [19]	24 CC nurses, four focus groups, two hospitals in Durban, South Africa	Second jobs via agency working	Agency working in by public sector nurses in ICU, private sector hospitals	(a) Demand for CC nurses in the private hospitals; (b) Salaries failing to keep pace with inflation; (c) Testing the experience of working in private sector	Reported that ICU s in private sector hospitals totally dependent on agency moonlighters-so staffed by ‘strangers’	(a) Reported tiredness, more likely to go off work sick; (b) Risk of ‘habitual moonlighting’; (c) Psychosocial problems- different mix of ethnicity and language in private hospitals	Private sector hospitals should develop clinical guidelines to ensure safety in hands of ‘strangers’
Brown (1999) [47]	In 1996, female registered nurses (RNs) were moonlighting at the rate of about 12%; female advanced practice nurses (APNs) were moonlighting at the rate of about 24%.	Second jobs held concurrently to the primary one; But also, second job not held continuously, i.e. irregularly.		On average, both RNs and APNs earn more on their second job then they do in their primary jobs. - The reason for the high rates of second-job holding among nurses relative to females in the general workforce appears to be a function of the nursing profession itself.	Those with second jobs tend, on average, to work fewer hours per week on their primary jobs than those who only hold one job.		
Creegan et al. (2003) [36]	51.7% of nurses in Australia worked part-time in 1997	Casualisation and non-standard (part-time) forms of nursing profession	Australia and references to the UK	(a) Women are more likely to work on a part-time basis in Australia. Nursing is a predominantly female profession; (b) Casualization is particularly common among Licenced Practical Nurses (16.5%) (c) Casualization is more common for rural States (20%)	(a) The hiring hospital has little control on quality and qualifications of agency nurses, used to top-up existing staff; (b) The savings associated with casualization of nursing are potentially large	Casual nurses do not enjoy the same protection and support systems as permanent ones, nor opportunities for professional development	(a) Need to understand better the shifting workforce patterns to improve management and planning of the nursing workforce; (b) To date, efforts have been directed towards recruiting more nurses in permanent positions. Better management of the casual workforce segment could represent an alternative policy.
Erasmus N. (2012) [53]	Broad focus on internship, unpaid overtime, moonlighting in SA. Mainly discusses doctors						Install electronic time recording in state hospitals, cessation of unpaid overtime, limits on medical intern shifts
Farzianpour et al. (2015) [61]	31% of nurses in private sector had a secondary job		Iran (Tehran private hospitals)				
Feysia et al. (2012) [62]	Repeating findings from Serra et al. (2010)			Repeating findings from Serra et al. (2010)			
Gillen (2013) [45]	1200 nursing magazine readers. 54% took up extra nursing work, and 10% another on outside nursing		UK, among Nursing Standard readers	To complement insufficient public sector salary 46% was thinking of leaving nursing profession		45.8% declared considering leaving nursing because poor salary	
Gupta and Dal Poz (2009) [41]	(a) 11% Chad; (b) 7% Cote d’Ivoire; (c)26% Jamaica; (d) 1% Mozambique; (e) 0% Sri Lanka; (f) 7% Zimbabwe	Work at another location (health facility or other) in the previous month	In some cases it was reported that nurses did not have the right of private practice				
Hipple (2010) [46]	(a) Report on data from US Labor stats; (b) 6.3% of nurses had multiple jobs- higher for male nurses (9.5%, but small sample)	Second jobs were in or out of health sector			Discusses possible impact of nurses working 12 h shifts- meaning they would have more free days for second jobs		
Knauth (2007) [32]	64% of nurses on 12 h nightshift and 45% of those on dayshift had a secondary job	Extended periods of work across different professions (Moonlighting and secondary jobs)	Nurses in 12 h night and day shifts		Increased sleepiness, difficulty of communicating with managers, increased risk of accidents driving home, increased absenteeism, reduced alertness		
Lane et al. (2009) [50]	A broad based global review of nursing labour market, health systems and macroeconomic policy	Has a brief section on dual practice as a ‘coping mechanism’	‘Dual practices range from legitimate private practice to moonlighting and informal charges for patients.’		Dual practice ‘often results in conflict of interest, idleness and absenteeism. However, not all dual practices can be considered as corrupt or leading to predatorial behaviour, but the impact of these practices can significantly undermine health services provision and public trust’.	‘Abusive multi-employment can result in negative consequences for health workers as well as patients’.	
MacLeod et al. (2017) [35]	1.1% of all nurses were in a job-share arrangement	(a) 15.8% of all nurses were in casual jobs; (b) Casualization is particularly common among Licenced Practical Nurses (16.5%); (c) Casualization is more common for rural States (20%)	Canada, Registered and Licenced nurse population in rural areas and in the north of Canada				Casualisation of nursing in rural areas/communities is seen as a concern that should be addressed by Govt policies
McPake et al. (2014) [17]	Reports examples and prevalence of NDP for several countries from secondary sources	Unspecified multiple job-holding	Low- and middle-income countries		Depending on its prevalence and regulation, dual practice can hamper attainment of UHC		Different regulatory option depending on country GDP, regulatory capacity, and definition of boundaries between public and private sectors
Monteiro et al. (2012) [40]	26% (out of 570 nursing workers)	Second job in the same branch	(a) Public hospitals and health centers in Brazil; (b) Nurses, nursing assistants and nursing auxiliaries				
Montour et al. (2009) [49]			Rural and community hospitals in the Hamilton Niagara area (Canada and US)	It was reported by nurses that part-time and casual nurses often seek employment in other hospitals and long-term care homes to supplement their income	Employment in multiple organisations contributes to scheduling issues because casual nurses are unavailable to fill vacant shifts		
Paina et al. (2014) [63]	Five public sector ‘facility case studies’ in Kampala Uganda	‘Additional jobs’ (Focus was on all workers, not just nurses)			‘Distinct challenges’ for local management in trying to manage internal dual practice opportunities, linked to opportunities to be involved in externally funded research projects within the hospital.		Variation between national – formal policy and local- invormal policy on allowing workers to have second jobs.
Portela et al. (2004) [39]	41.5% of public hospital nurses were moonlighters	MJH was more common among nurses during 12 h night or day shifts	Public hospitals in Brazil			Long (12 h) night shifts found to have a less taxing effect on nurses health than day ones.	
Ribeiro-Silva et al.(2006) [51]	33%	Second job in another hospital or clinic	(a) Public hospitals Brazil; (b) Registered nurses and nursing assistants (*N* = 144)		Those who had two jobs devoted more time to sleep/rest on the job	(a) The less time devoted to leisure and personal needs among those who work more associated with quality of daily life; (b) Sleep on the job related to working on a second job. Those with two jobs had longer sleep episodes on the jobs (compared with those with a single job)	
Rispel and Blaauw (2015) [21]	40.7% reported agency or moonlighting in previous year	Agency/ moonlighting	80 hospitals in 4 provinces in SA. All nurses surveyed		11.9% of moonlighters had taken vacation to do agency work or moonlight; 9.8% reported conflicting schedules between primary and secondary jobs		Strong nurse leadership, effective management and consultation with front line nurses to counteract potential negative impacts of agency- moonlighting
Rispel et al. (2011) [64]	(a) 28.0% IC95 [24.2; 32.1] moonlighting last 12 months; (b) 42.2% moonlighting ever; (c) 37.8% IC95 [32.4;43.6] agency nursing; (d) 69.2% IC95 [64.1;73.8] had done overtime, moonlighting or agency nursing in the preceding year; (e) 18.5% reported all activities	Additional paid work (nursing or not nursing nature) in private health facility, another government health facility, insurance company private health laboratory or same health care facility excluding overtime		(a) Taking care of patients, opportunity to learn new nursing skills, relationship with co-workers, agency’s weekly pay, choice of unit/ward, job variety, do it for the money, stimulating work, quality of supervision, modern equipment/infrastructure, selection of working hours, money owed to revenue service; (b) Predictors: - having children province, sector (higher among private sector) - professional nurse vs nursing assistant/ auxiliary nurse - working in adult critical care unit vs paediatric critical care unit. Working in general wards and other wards protects for moonlighting compared to working in paediatric critical care unit			Management of moonlighting was considered an important policy priority by the SA Government which is reflected in the 5-year HRH strategy
Rispel et al. (2014) [33]	28% moonlighting (965/3442)	Additional paid work (nursing or not nursing nature) in private health facility, another government health facility, insurance company private health laboratory or same health care facility excluding overtime; Last 12 months	–	–	(a) Intention to leave was higher among moonlighters compared to non-moonlighters; (b) Planning to go overseas was higher in moonlighters compared to non-moonlighters; (c) Moonlighting is a predictor or intention to leave primary health job	–	–
Salmon et al. (2016) [25]	(a) Report of a Bellagio meeting ‘focusing on the largely overlooked area of investment in nursing and midwifery enterprise as a means for both empowering women and strengthening health systems and services’	Second jobs only indirectly examined in the broader context of the objectives of the meeting.					
Seleghim et al. (2012) [65]		Simultaneous other job (not specified) for 15% of nurses (just 5…)	Paraná State, Brazil		No specific health consequence, but sample too small for significance		
Serra et al. (2010) [34]	(a) 5% of all the nurses followed had secondary jobs; (b) Average days per week in secondary job was 9 days. Average hours per day was 9 h (Vs 9.9 in primary)	87% of moonlighting nurses had a full-time job in public sector, and a secondary job in private/NGOs	Ethiopia, urban and rural areas	A greater proportion of nurses (50%) agreed in the second wave that you need to take up a secondary job to earn enough to support families			
Stephenson (2017) [37]	Of 900 nurses completing an online survey in the UK, 47% declared engaging in bank and/or agency shifts	Ban on agency shifts is introduced for nurses with a substantial NHS contract as a cost-containment strategy for public sector	The UK	Agency and bank shifts as a way to balance the cap on increasing public sector salaries			
Tailby (2005) [43]	(a) 80% (of 185,000) nurses registered with NHS nurse banks had another nursing job; (b) 60% worked occasionally or regularly additional shifts paid at bank rates or agency rates	–	–	(a) Need for additional income; (b) Need to refresh/update skills; (c) Attain a preferred pattern of working hours	–	–	–
Taylor et al. (2004) [52]		Casualisation of work	Mental health nurses in New South Wales, Australia			Casualisation of work was reported to be a major source of career fatigue and burnout	
Wynton and A. Kleebauer (2016) [38]		Agency nursing, other country (England, Scotland)		Economic- higher rate for single day fees than in home country of N Ireland			

For Australia, Creegan et al. [36] show that 51.7% of nurses worked part-time in 1997, while in 2011 Batch and Windsor found that the nursing profession had a higher rate of casualization than other professional and highly skilled workforces, and that 47.5% of nurses were employed in non-standard work [44].

For the UK, Tailby reports that 80% (of 185 000) nurses registered with NHS nurse banks had another nursing job, and 60% worked occasionally or regularly additional shifts paid at bank or agency rates [43]. In a survey among nursing magazines’ readers in 2013 [45], 54% declared taking up extra nursing work, and 10% another full time job outside nursing; 5 years later, 47% of the 900 nurses participating in another online magazine readers survey declared engaging in bank and/or agency shifts [37].

A report from the US Bureau of Labor Statistics [46] shows that multiple job-holding has grown steadily over the last decades, that 6.3% of nurses had multiple jobs in 2014, and that such prevalence was higher for a small sample of male nurses (9.5%). A 2017 article from Canada [35] provides evidence that 15.8% of all rural nurses are in casual jobs and that casualization is particularly common among registered nurses and licenced practical nurses (16.5%), and more common among those nurses living in the north of the country (20.0%).

Evidence on the phenomenon from LMICs is substantial too; Gupta et al. report from a multi-country study that nurses dual practice would be more limited than physicians’—the former calculated to be 11% in Chad; 7% in Cote d’Ivoire; 26% in Jamaica; 1% in Mozambique; 0% in Sri Lanka; and 7% in Zimbabwe [41]. In a World Bank study in Ethiopia [34], a similar proportion of a cohort of public sector nurses (5%) were found to have secondary jobs 5 years after their initial appointment. Several studies by Rispel and colleagues from South Africa showed the prevalence of different forms of multiple employment to be common (around 28%) and on the rise among South African nurses [21, 33]. And in Brazil, Portela et al. showed 41.5% of nurses in two public hospitals to be moonlighters [39].

### Drivers and motivation

A few individual and institutional drivers for the practice are recurrent in the literature. At a personal level, the need to increase overall earnings by supplementing income from main salary is by far the most common, such as in Northern Ireland and elsewhere in the UK—where holding multiple jobs is seen by many nurses as an essential way to increase income [38, 45]. However, also for a low-income country like Ethiopia where a nurse’s salary is typically higher than the country’s average Gross Domestic Product (GDP) per capita, Serra et al. report that half of the nurses followed in their study took up a second job to support their families [34].

Flexibility of additional part-time employment seems to be another key factor for Australian and UK nurses, since nursing is a typically female profession, and some female workers have a strong preference for part-time, flexible jobs in comparison to their male peers [36, 43]. In the surveys in South Africa [21, 33], the opportunity for learning new nursing skills, the need to introduce diversity in professional routines, a more stimulating working environment, the quality of supervision, and the ability to select their own working hours were the key reported motivating factors for South African nurses.

At a more institutional level, also in South Africa, the growing demand for nursing services from the private sector is pointed to as the key driver of the phenomenon of casualization of nursing employment. Taking a broader organisational perspective, Batch and Windsor (2014) argue that the ‘casualization movement’ is really aimed at creating a more flexible, cheaper, and easier to manage the nursing workforce.

### Consequences of nurses’ multiple job-holding

No specific study appears to have assessed the impact of nurses’ dual practice, although many articles offered hypotheses and interpretations in regard. Generally, health worker’s dual practice is regarded unfavourably in the academic literature. McPake et al. argue that, depending on its forms and prevalence, it could hamper the attainment of UHC in some countries [2]. Others report that the associated increased tiredness and lack of alertness for casual workers who work long hours in multiple jobs, as well as their difficulty of communication with resident staff, are reported to substantially increase the risk of clinical accidents [32]. However, a PhD dissertation work from the USA shows that, on average, nurses with a secondary job tend to work fewer hours in their primary, public employment than their non-moonlighting colleagues [47]. Studies in South Africa suggest that moonlighters are also more likely to take vacation and time out from their main employment to pursue other jobs [21], and intentions to leave the public sector and/or migrate have been found to be more frequent among them than in their single-job peers [33].

Baumann et al. argue that employing an unbalanced proportion of full-time and casual nurses reduces flexibility of a hospital management, as these latter would be less available to cover for unforeseen needs [30]. In the case of Ontario, Canada’s experience with the Severe Acute Respiratory Syndrome (SARS) epidemic, such factors, together with the increased dependence of many hospitals in high-income countries on agency nurses, have been suggested could compromise the system’s ‘surge capacity’, that is, its ability to rapidly scale-up services and response in the face of epidemics [48]. In a qualitative study in rural community hospitals in Canada about the changing nature of nursing work, Montour et al. argue that employment in multiple organisations contributes to scheduling issues because casual nurses are unavailable to fill vacant shifts [49]. And finally, according to some studies, some types of health workers’ dual practice can critically undermine health service provision and public trust, as it often entails conflict of interest, idleness, and absenteeism [50].

At a more personal level, Portela et al. show that taking extra shifts can seriously affect nurses’ general health and exhaustion levels, with night shifts reported to be less disruptive than day ones [39]. Such findings on sleeping patterns are also echoed by Ribeiro-Silva et al. [51] for hospital nurses in Rio de Janeiro, Brazil, and by Knauth for workers outside the clinical profession [32]. Casualization of work was also identified as a major source of career fatigue and burnout in qualitative interviews with nurses in Australia [52]. Marginalisation and exclusion of part-timers by their peers was also reported to be a major source of dissatisfaction and frustration in an ethnographic study on Australian nurses [44].

As a positive individual consequence, nurses were reported to value highly the opportunity dual work offers to complement meagre public salaries in high-income countries [45] and to support extended families in low-income ones [34]; in the USA in 1999, nurses earned more in their secondary job than in their primary employment [47]. Flexible working hours is another characteristics that nurses would find particularly attractive in secondary, casual jobs in the UK [43]. In this respect, Creegan et al. suggest that flexible working arrangements would be particularly suited for the predominantly female nursing workforce [36].

### Policy options

Only a minority of the studies retrieved in this review (eight) present and discuss possible policy options for managing, regulating, or controlling the practice. McPake et al. [2] link the choice of policy measures to the prevalence of the practice and to the country’s regulatory capacity. Rispel et al. [21] highlight managing moonlighters as a key human resource for health strategy in South Africa; consultation with frontline nurses to counteract the practice’s negative impact is suggested as a possible policy option.

Electronic time recording, cessation of unpaid overtime, and controls over the number of shifts are put forward as alternative measures by other authors [53], while developing clinical guidelines for hospitals to ensure safety of services ‘in the hands of strangers’ has been called for as a possible institutional measure. Other scholars have argued for the need for a better understanding of dual practice patterns, in recognition of the fact that more effective planning and management of a flexible workforce could represent a more suitable solution than prohibition [36].

## Discussion

Our review revealed that nurses engage in multiple job-holding activities, with varying forms and prevalence in high-income as well as in low-income countries. The practice appears to be driven by multiple, complex, and varying factors beyond the obvious economic motif, and to have non-trivial consequences, particularly for nurses’ welfare, organisation of health services, and health labour market. Despite its prevalence and relevance, a surprising paucity of studies was found on nurses’ dual practice, and very few policy options have been outlined in the literature to address the phenomenon.

Although in the nursing profession holding simultaneously multiple jobs cannot be necessarily considered as dual practice, the two areas often overlap, in shapes of poorly demarcated contours. Consistently with what is observed for other professions, more than one way seems to exist for nurses to engage with dual practice, both in regulated and informal, casual fashions. This may at least in part explain why the practice has been under-reported and little regulated through the years, with some of its forms driven underground or even considered illegal in some countries [34], and other forms—such as the ‘casualization’ of nursing services—only recently having come to the fore in the context of rapidly evolving health labour markets [33]. This absence of usable datasets would call for primary research to be conducted to, first, explore through qualitative research the specificities of the phenomenon and, second, to measure them quantitatively.

Unsurprisingly, our review of the available evidence appears to show that economic considerations are not the sole driver for nurses taking on simultaneous multiple jobs [33, 54]. A basic dissatisfaction with the limited range of duties performed in their main job, limited opportunities for development, or availability of time made possible by night shift arrangements, are other important factors that may help explain such a decision. Although much effort has been devoted in the past to understanding nurses’ burnout [55, 56], surprisingly little attention has been given to the tendency to take on additional work in presence of an already heavy workload.

In contrast to the comparatively better understood physician dual practice, the limited evidence reviewed suggests that nurses’ dual practice is more likely to be bounded by the very nature of their jobs than it is for physicians, as typically nurses have limited autonomy and tend to work as part of a team, rather than as individual providers. On this basis, a hypothesis could be made that, while nurses are more likely to be part of an established team in their main (public sector) job, second jobs are often taken up as individuals, as agency nurses for one shift, or private home care visits.

Nurses’ personal characteristics also appear to shape forms and extent of the practice in any one country. Since taking up additional work in the private sector may be financially rewarding, but will also add to overall workload and may not necessarily increase career prospects, younger and comparatively lower paid nurses seem to be the ones likely to engage more in the practice [35, 39]. As a compounding factor, as nurses are predominantly female and often perform a disproportionate share of child-rearing and care for elderly or disabled relatives’ duties, we may speculate that having dependents will likely decrease their ability to take up additional hours, unless the additional income generated can compensate for any additional child care costs.

The evidence available suggests that the consequences of this phenomenon are not negligible, particularly for the health of those nurses ending up working longer hours and hospital shifts because of their multiple commitments [39, 51], but also for the organisation of public and private health services facing a more ‘casual’ and less-committed kind of workforce [21]. Interestingly, the most recent literature on nursing and midwifery enterprises [24, 25] recognises this limitation and may lay the grounds for a different type of engagement of nursing staff with private sector activities. We also did not find any evidence regarding the importance of economic considerations of nurses’ dual practice, or of any difference between higher and lower income countries; as we suspect the implications of such practice may have substantial repercussions on the health labour market, this could represent an area of future research.

This paper is based on a scoping exercise and so has limitations. The limited and often incomplete evidence made it difficult to be certain if dual practice is a factor of relevance in all health systems worldwide, if it is a major issue for nurse labour market participation, and its overall impact on the provision of care. With respect to the latter, this may be because some aspects of dual practice are on the margins of ‘formal’ work and may go unrecognised by formal systems of employment and regulation.

All of the above call for a deeper understanding of the phenomenon, with the objective of better harnessing the changing nurses’ workforce worldwide. Following our review, the core elements of the required research agenda on nurse dual practice appear to be three-fold. First, further research is needed to systematically explore the nature, extent, and impact of nurse dual practice in different systems and countries; this can be achieved through the analysis of employment and professional register/association data sets where these exist, or by ad-hoc surveys of nurses and/ or workplaces. Analysis of specific data sets in some countries (e.g. such as the Current Population Census [57] and the Integrated Public Use Microdata Series the United Sates; Labour Force Surveys; and professional registries) may provide more evidence on prevalence of dual practice and some of its main forms.

Secondly, there is a need for developing a more informed picture of the reasons why nurses take on dual practice, their experiences and preferences of dual practice, and the impact on their broader work/life balance. This can be achieved through a qualitative approach, exploring multiple contexts in high- and low-income settings, and different nursing profiles.

Finally, there is a gap of research that establish the impact of dual practice at the policy level—what is its impact on participation rates, overall nursing hours available in different systems, what are the trends in incidence, what is the impact on nurses, and on the quality of care that is being delivered. Measures could be needed to mitigate the effects of nurses’ dual practice to protect the provision of free-of-charge public sector for vulnerable populations. This latter area for policy research is the most complex and challenging to interrogate, but also of potentially great significance. Without a better understanding of nurse dual practice, it will continue to be a largely ‘hidden’ element in nursing workforce policy and practice, with an unknown level of significance, and an unclear impact on the delivery of care.

## Additional files

Additional file 1: Search terms—database searches. (DOCX 19 kb)

Additional file 2: Spreadsheet of papers reviewed. (XLSX 32 kb)

## Abbreviations

CINHAL: Cumulative Index to Nursing and Allied Health Literature
EBSCO: Elton B. Stephens Co.
GDP: Gross Domestic Product
HRH: Human Resources for Health
ISI: Institute of Scientific Information
LMIC: Low- and middle-income countries
SARS: Severe acute respiratory syndrome
USA: United States of America
UHC: Universal Health Coverage
