# Supplementary material for: Understanding nurses’ dual practice: a scoping review of what we know and what we still need to ask on nurses holding multiple jobs
Source: Hum Resour Health. 2018 Feb 22;16:14. doi: 10.1186/s12960-018-0276-x (PMC5824568; doi:10.1186/s12960-018-0276-x)
Supplement: Supplementary file 1 — Search terms—database searches. (DOCX 19 kb) [file 12960_2018_276_MOESM1_ESM.docx]

**Additional file 1**

SEARCH TERMS – DATABASES SEARCHES

**PUBMED**

("Employment/supply and distribution"[Mesh] OR "Employment/manpower"[Mesh] OR

"dual practice*" OR "moonlight*" OR "Dual job*" OR "second job*" OR "casualization of work" OR "casualisation of work") “temporary employment” and “multiple employers” **AND** ("Nurses"[Mesh] OR "Nurs*" OR "Licensed Practical Nurses"[Mesh] OR "nursing"[Subheading] OR "nursing"[Mesh] OR "Nursing Staff"[Mesh])

**ISI Web of knowledge**

(TS=(nurs*) OR TI=(nurs*)) **AND** (TS=(moonlight*) OR TS=("Dual job*") OR TS=("dual practice*") OR TS=("second job*") OR TS=("casualization of work") OR TS=("casualisation of work"))

**Scopus**

TITLE-ABS-KEY (nurs*)  **AND** TITLE-ABS-KEY (moonlight*)  OR  TITLE-ABS-KEY (“dual  practice*“)  OR  TITLE-ABS-KEY (“second  job*”)  OR  TITLE-ABS-KEY (“dual  job*”) OR  TITLE-ABS-KEY ("casualisation of work") OR  TITLE-ABS-KEY ("casualization of work")

**CINHAL**

## (TX nurs* OR TI nurs* OR AB nurs*) **AND** (TI "causalization of work" OR AB "causalization of work" OR TI "causalisation of work" AB "causalisation of work" OR TI "moonlight*" OR AB "moonlight*"  OR TI "dual practice*" OR AB "dual practice*" OR TI "dual job*" OR AB "dual job*" OR TI "second job*" OR AB "second job*")

**SEARCHES FOR THE GREY LITERATURE**

World Health Organization

Specifically searched: http://www.who.int/hrh/nursing_midwifery/documents/en/

International Labour Organization (http://www.ilo.org/global/lang--en/index.htm)

Alternative keywords inserted into the search tool: nursing; dual practice nursing; second job

International Council of Nurses

Specifically searched: [International Centre for Human Resources in Nursing publications](http://www.icn.ch/pillarsprograms/ichrn-publications/) (http://www.icn.ch/publications/free-publications/)

Global Health Workforce Alliance (Knowledge centre: http://www.who.int/workforcealliance/knowledge/en/)

Themes specifically searched: Career choice; Private sector; labour markets

Canadian Nurses Association (https://www.cna-aiic.ca/en)

Alternative keywords inserted into the search tool: second job; dual practice; moonlighting

American Nurses Association - publications (http://www.nursingworld.org/HomepageCategory/ANAPublications)

Search for: second job; dual practice; moonlighting

Australian Nursing and Midwifery Federation

Reports: <http://anmf.org.au/pages/anmf-reports>

Media releases: <http://anmf.org.au/media-releases>

Royal College of Nursing library,

Alternative keywords inserted into the search tool:: second job; dual practice; moonlighting

- excluding peer-review scholarly papers

**Contacts with Experts as Key-Informants**

The following experts were contacted via email in March 2017 on the identification of relevant references:

Jim Campbell, (World Health Organization)

Mário Dal Poz, Instituto de Medicina Social, Universidade Estadual do Rio de Janeiro (Brazil)

Gilles Dussault, Instituto de Hygiene e Medicina Tropical, Universidade Nova de Lisboa (Portugal)

Howard Catton, International Council of Nurses, Geneva (Switzerland)

Laetitita Rispel, University of Witswatersrand (South Africa)

Marla Salmon, University of Washington (USA)

Linda Aiken, University of Pennsylvania (USA)

Ann Marie Rafferty, Kings College (UK)
